# Safety Parameters for the Use of Holmium:YAG Laser in the Treatment of Biliary Calculi: The Ex-Vivo Model

**DOI:** 10.3390/medicina60020346

**Published:** 2024-02-19

**Authors:** Sandeep Patel, Dustin Kiker, Utpal Mondal, Hari Sayana, Shreyas Saligram, Laura Rosenkranz, Samuel Han

**Affiliations:** 1 Division of Gastroenterology and Nutrition, UT Health San Antonio, San Antonio, TX 78229, USA; patels7@uthscsa.edu (S.P.); dkiker@tddctx.com (D.K.); umondal2002@yahoo.com (U.M.); sayana@uthscsa.edu (H.S.); drsaligram@yahoo.com (S.S.); rosenkranz@uthscsa.edu (L.R.); 2 Division of Gastroenterology, Hepatology, and Nutrition, The Ohio State University Wexner Medical Center, Columbus, OH 43210, USA

**Keywords:** laser lithotripsy, choledocholithiasis, cholangioscopy, holmium

## Abstract

*Background and Objectives*: While studies have demonstrated the efficacy of cholangioscopy-guided Holmium-Yttrium aluminum garnet (Ho:YAG) laser lithotripsy for the treatment of refractory bile duct stones, data regarding the safety of the operating parameters for laser lithotripsy are lacking. The aim of this study was to determine safe, yet effective, energy settings for Ho:YAG laser in the ex-vivo model. *Materials and Methods*: This ex vivo experimental study utilized the Ho:YAG laser on porcine bile duct epithelium and human gallstones. Ho:YAG laser lithotripsy was applied in different power settings from 8 to 15 Watts (W) to six explanted porcine bile ducts. Settings that appeared safe were then utilized to fragment seventy-three human gallstones. *Results*: The median bile duct perforation times with the Ho:YAG laser between 8–15 W were: >60 s (8 W); 23 s (9 W); 29 s (10 W); 27 s (12 W); 12 s (14 W); and 8 s (15 W). Statistically significant differences in the median perforation times were noted between 8 W vs. 15 W, 9 W vs. 15 W, 10 W vs. 15 W, and 12 W vs. 15 W (*p* < 0.05). When using a 365 µm Ho:YAG laser probe at 8–12 W, the fragmentation rates on various size stones were: 100% (<1.5 cm); 80–100% (1.6–2.0 cm) and 0–32% (>2.0 cm). Optimal fragmentation was seen utilizing 12 W with high energy (2.4 J) and low frequency (5 Hz) settings. Using a larger 550 µm probe at these settings resulted in 100% fragmentation of stones larger than 2 cm. *Conclusions*: The Ho:YAG laser appears to be safe and effective in the treatment of large bile duct stones when used between 8–12 W in 5 s bursts in an ex vivo model utilizing porcine bile ducts and human gallstones.

## 1. Introduction

Choledocholithiasis is a prevalent and costly health care issue worldwide. Although initially a surgically-managed disease, the majority of the retained bile duct stones can now be resolved using standard techniques employed via endoscopic retrograde cholangiopancreatography (ERCP) [1]. Approximately 10–15% of bile duct stone cases prove refractory to conventional endoscopic extraction methods, such as balloon sweeping or basket removal [2]. These refractory stones can include large (>10 mm) stones, intrahepatic stones, or impacted stones [3]. These can be particularly challenging to remove given the relatively small size of the bile duct orifice in comparison to the size of the stone or in the presence of a biliary stricture where stone clearance requires stricture treatment as well. Additionally, in cases of impacted stones, the inability to maneuver devices past a stone severely limits any form of lithotripsy attempts.

Over the years, several techniques have been developed to manage refractory bile duct stones with varying success, including mechanical lithotripsy, endoscopic papillary large balloon dilatation, and intraductal lithotripsy with either electrohydraulic lithotripsy (EHL) or laser lithotripsy (LL) with pulsed dye lasers [4,5,6,7,8,9,10,11]. Several randomized studies have consistently demonstrated >90% success rates in complete stone clearance using LL for large or refractory bile duct stones [5,6,12,13]. A single-center randomized trial (n = 60) comparing cholangioscopy-guided LL with papillary balloon dilation/mechanical lithotripsy demonstrated a significantly higher success rate with LL in stone clearance (92.9% vs. 67%) for stones >1 cm in diameter [5]. This was mirrored in another single-center randomized trial (n = 66) comparing LL with papillary balloon dilatation which found not only a higher stone clearance success rate with LL (93.9% vs. 72.7%) but also that treatment success was associated with a stone to extrahepatic bile duct diameter ratio ≤1 as well as the absence of a tapered bile duct [12]. A two-center study (n = 32) compared LL with mechanical lithotripsy for the treatment of large bile duct stones that were unable to be removed with papillary balloon dilation [6]. They similarly found a high stone clearance rate (100% vs. 63%) while also noting that LL enabled complete stone clearance in all patients in a single session. Lastly, a single-center randomized trial (n = 157) compared cholangioscopy-guided LL with laparoscopic common bile duct exploration for very large bile duct stones (≥2 cm) [13]. This study found cholangioscopy-guided LL to be non-inferior to laparoscopic stone extraction with no difference in adverse events between the 2 treatment groups. Notably, patients treated via cholangioscopy-guided LL had a shorter length of stay and were found to have a greater quality of life at the 1- and 3-month follow-ups than those treated via surgery. Based on these data, cholangioscopy-guided LL appears to offer an effective treatment option for refractory bile duct stones.

**Figure 1:**
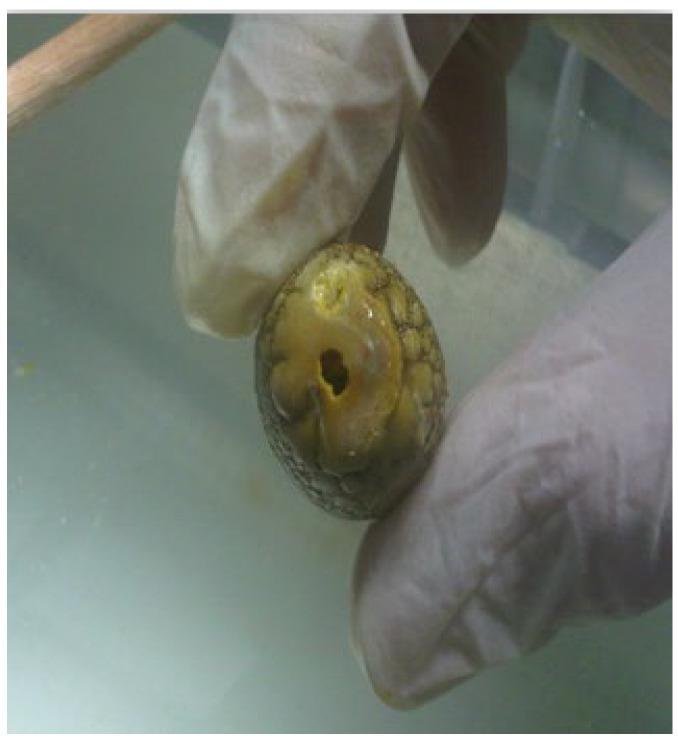
Example of drilling effect where a hole is bored into the stone without significant fragmentation.

Despite its increasing applications including use with pancreatic duct stones, there remains limited data as to the safety parameters and ideal operating settings of this technology for refractory bile duct stones. In particular, one of the most prevalent obstacles to stone extraction using the Ho:YAG laser has been the propensity to bore holes through the stone without actually causing meaningful fragmentation, a phenomenon we term as ‘drilling’ (Figure 1) [14].

The primary aim of this study was to establish safety data as well as operating parameters for the use of the Ho:YAG laser in the bile duct using a porcine model. We also aimed to determine the optimal power settings required to effectively fragment stones within the determined safe power settings, while avoiding the drilling effect.

## 2. Materials and Methods

This study received exemption from the Institutional Animal Care and Use Committee at the UT Health Science Center at San Antonio as all specimens had been previously harvested.

### 2.1. Safety Phase

**Figure 2:**
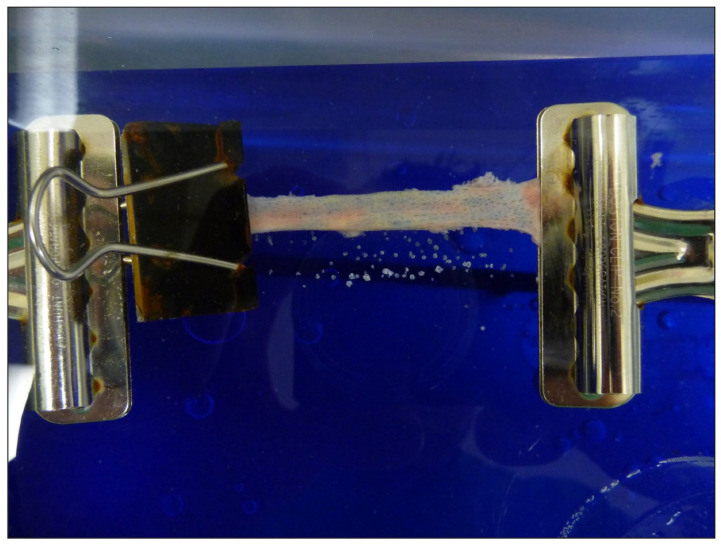
Excised bile duct submerged in saline for safety testing.

To determine safe operating parameters within the bile duct, we utilized harvested bile ducts from 6 female 50 kg domestic Yorkshire pigs. Each bile duct was excised longitudinally to expose the epithelial surface and was then submerged in a holding device under tension in a liquid saline media (Figure 2). A Slimline Ho:YAG 365 µm laser probe (Lumenis, Sunnyvale, CA, USA), which emits at a wavelength of 2100 nm, was directly placed (without the use of an endoscope) in perpendicular contact with the ductal epithelium and activated at various energy density settings to evaluate the time needed to cause perforation as well as the overall perforation rates for each setting. The power settings ranged from 8–15 Watts (W) and were accomplished using various combinations of frequencies (Hz) and Joules (J). The laser was activated until perforation of the bile duct occurred or for a maximum of 60 s.

For each setting, the probe was placed in similar locations on both the proximal and distal ducts. The location and wattage were randomized to avoid physiologic differences that could impact on outcomes. Overall, 104 data points were collected using 6 different power settings (8 W (n = 22); 9 W (n = 22); 10 W (n = 16); 12 W (n = 20); 14 W (n = 8); 15 W (n = 16)).

### 2.2. Fragmentation Phase

We applied the results of the 1st phase of this study to 73 biliary stones retrieved from post-cholecystectomy patients, ranging in size from 0.6–3.3 cm. The individual stones were carefully measured and their constituency characterized as cholesterol, pigmented, or mixed based on their gross appearance. Each stone was placed in a plastic tube to simulate the bile duct and submerged in a saline medium. A 365 µm Ho:YAG laser probe was placed within 1–3 mm of the stone and then activated. The power settings used ranged from 8–12 W and were accomplished using various combinations of frequency (5–20 Hz) and Joules (0.4–2.4 J). The laser was activated in five second bursts until stone fragmentation was achieved or until a maximum of 60 s had elapsed. Fragmentation was defined on a scale of 1–4, based on the amount of stone fragmentation that occurred: (1) Complete fragmentation without drilling; (2) Slight drilling with fragmentation; (3) Cleavage into 2 pieces after heavy drilling effect; (4) Complete drilling without any fragmentation. Successful fragmentation was defined as reaching a score of 1, 2, or 3 on the scale, whereas failure was defined as a score of 4.

After the initial data analysis was complete on the first 73 stones, 12 additional large biliary stones ranging in size from 2.1–4.5 cm were then evaluated using the same process, but with a larger 550 µm Slimline Ho-YAG laser probe and targeted power settings of 12 W at a low frequency of 5 Hz. The initial scoring scale was used, and the results compared to those obtained when using the smaller 365 µm probe at equivalent power settings as well as against all previous power settings used on stones larger than 2 cm.

### 2.3. Statistical Analysis

For the first aim of this study, the median perforation times at each power setting were compared using the Kruskal-Wallis One Analysis of Variance to determine the settings that proved safe for use in the bile duct while having the least potential for perforation. Pairwise multiple comparisons by Dunn’s Method were performed to compare the median perforation times between different energy settings.

For the second aim of the study, variables including stone constituency, size, laser power, and frequency were analyzed using ANOVA with a covariance analysis to assess predictive factors for effective stone fragmentation.

## 3. Results

**Figure 3:**
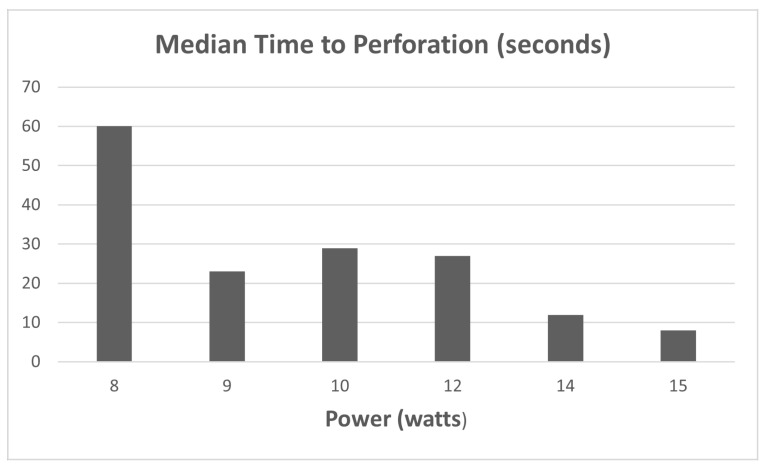
Comparison of stone fragmentation rates by laser frequency.

From the 104 data points collected during the 1st phase of the study (Ho:YAG laser safety in the bile duct), the median perforation times were as follows as seen in Figure 3: 8 W (>60 s); 9 W (23 s); 10 W (29 s); 12 W (27 s); 14 W (12 s); and 15 W (8 s). Statistically significant differences in median perforation times were noted between 8 W vs. 15 W, 9 W vs. 15 W, 10 W vs.15 W, and 12 W vs.15 W (*p* < 0.05). There was no statistical difference between 14 and 15 W.

From the 73 biliary stones subjected to ex vivo fragmentation using the 365 µm Ho:YAG laser probe in the 2nd phase of this study, laser power setting and stone constituency were not significantly associated with stone fragmentation. There was a statistically significant negative association between stone size and fragmentation as the ability to fragment stones diminished with increasing stone size (*p* < 0.05). There was also a significant negative association between laser frequency and stone fragmentation, as the ability to fragment stones decreased with increasing laser frequency (*p* < 0.05). There was 100% fragmentation of stones < 1.5 cm regardless of frequency setting. For stones between 1.6 and 2.0 cm, successful fragmentation was achieved in 100% of the stones at the lowest frequency (5 Hz) but only in 80% of stones at the highest tested frequency (20 Hz). Stones > 2.0 cm presented a challenge at every setting as fragmentation was achieved in only 32% at the low frequency (5 Hz) and in 0 stones at the highest frequency (20 Hz).

When subjecting the additional 12 large stones (>2.0 cm) to energy settings that gave the most effective fragmentation (12 W at 5 Hz) using the Slimline 550 µm laser probe 100% fragmentation rates were achieved. This was a 67% increase in the ability to fragment large stones > 2.0 cm when compared to the same setting using the 365 µm probe (*p* < 0.05). There was also a significant difference in the fragmentation rates of stones >2.0 cm using the 550 µm probe at 12 W and 5 Hz vs. all other settings used on stones >2.0 cm with the 365 µm probe (*p* < 0.05).

## 4. Discussion

Choledocholithiasis occurs in 10–20% of patients with cholelithiasis and is responsible for over 10,000 hospital admissions/year [2,15]. Choledocholithiasis has largely become a non-surgical disease, with ERCP representing 1st-line therapy with standard techniques including biliary sphincterotomy and balloon and/or basket extraction [1]. While mechanical lithotripsy can increase stone extraction success rates to 90%, 10–15% of bile duct stone cases prove to be refractory to conventional endoscopic methods [16]. Barriers to stone extraction can include the altered anatomy of the bile duct or papilla, intrahepatic stones, stone(s) proximal to a stricture, and large stones.

For the management of difficult bile duct stones, current guidelines recommend performing endoscopic papillary large balloon dilatation after biliary sphincterotomy [1,17]. Should stone extraction still be unsuccessful, cholangioscopy-guided lithotripsy utilizing either EHL or LL is recommended for stone removal [1,17]. EHL accomplishes this by utilizing a high-energy electric source (50–150 W) delivered through a probe, which in a fluid-filled bile duct creates a cavitary bubble at its tip. The physical oscillations of this cavitary bubble generate shock waves, which cause fragmentation. Stone fragments produced with EHL tend to be large and sharp with adverse events such as hemobilia, cholangitis, pancreatitis, perforation, and bile leakage occurring at a rate of 3–15% [18,19]. LL also creates a cavitary bubble in an aqueous solution but utilizes a lower energy optical source. To this end, several early studies demonstrated the feasibility of utilizing a variety of lasers such as neodymium, coumarin green, rhodamine-6G dyes, and holmium to treat difficult bile duct stones [10,11,20,21].

Holmium is the newest of the lithotrites to be introduced for biliary stone fragmentation. Because the wavelength of holmium (2140 nm) is almost identical to that of water (1940 nm), the shockwave scatter generated in an aqueous solution is almost completely absorbed and incidental trauma reduced. Another favorable attribute is holmium’s relatively shallow depth of penetration (0.4 mm) on tissue, which is one-tenth that of the other lithotrites such as neodymium (>5 mm) [22]. On a practical level, this allows for effective stone fragmentation while minimizing damage to the surrounding biliary tissue. Low energy precision and decreased tissue penetrance give holmium-YAG laser theoretical physical safety advantages over EHL and the other laser lithotrites in this space.

Several randomized trials have now demonstrated the high success rates of LL using the holmium-YAG laser for the treatment of difficult bile duct stones, consistently finding success rates >90% [5,6,12]. Importantly, a single-center randomized trial found LL to be non-inferior to laparoscopic bile duct exploration while resulting in shorter hospitalizations and quicker recovery, further supporting the role of LL in cases of challenging bile duct stones [13]. These studies, however, provide limited data in regard to the operating parameters for the use of holmium-YAG laser, which our study aims to provide to aid the endoscopist in performing intraductal LL.

One of the major potential adverse events of using a laser in the bile duct is the penetration of the relatively thin wall of the duct and the causation of a perforation. Our data suggests that conservative use of holmium-YAG laser lithotripsy in the bile duct is safe within the parameters of 8–12 W even when up against the epithelium for up to 25 s. It is fair to say that it may yet be safe to use higher power settings or a longer interval of activation when the laser probe is in direct contact with a stone, and not the ductal epithelium. However, visualization of the tip of the probe can become limited with active laser fragmentation. We therefore recommend the above power settings in five-second activation intervals for the use of the holmium-YAG laser in the bile duct. This provides an ample safety margin (five seconds) for inadvertent contact with the bile duct epithelium. In line with this, LL is best performed using a cholangioscopy under direct visualization. Early studies examined LL using a flashlamp-excited dye laser and a pulsed dye laser under fluoroscopic guidance, finding that the cholangioscopy-guided application was more effective in the successful fragmentation of bile duct stones [23,24]. Specifically, investigators found it challenging to properly orient the laser probe using fluoroscopy alone, which speaks to the difficulty in ensuring that the laser is being directed at the stone and not the bile duct wall.

Within the safe power settings of 8–12 W, the major variables affecting stone fragmentation appear to be stone size and the frequency of the laser used. Although it did not prove statistically significant, an increased power setting affected the success of fragmentation, particularly in larger stones (>1.6 cm). While it seems that the power setting between 8–12 W initially chosen does not make a significant difference in stones of size 1.5 cm or less since all were successfully fragmented, the initial settings become more important with larger stones. This is in line with the above-mentioned randomized studies which in general utilized a 10 W setting. It appears that larger stones (>1.6 cm) fragment much better with lower frequency settings whereas a more significant drilling effect is encountered at higher frequencies. The ability to fragment stones >2 cm appears diminished at all settings but best results were seen at settings of 12 W and 5 Hz. This limitation was overcome by using a larger 550 µm diameter probe at this setting (12 W; 5 Hz).

It was noted that direct contact with the stone as well as pressure placed upon an impacted stone by an activated laser probe resulted in a greater drilling effect regardless of the setting used. It was also noted that once the probe would drill into a stone, and especially if it drilled through a stone, there was a propensity for the laser fiber to become embedded in the stone and thus difficult to remove. This phenomenon was previously explained when Schafer et al. noted that the effect of holmium laser on stones was due to both the direct melting effect of the stone material as well as the ejection of that melted material, as the acoustic bubble created at the tip of the fiber collapsed [25]. It was also noted that the melted material, if not immediately ejected, would resolidify. This may, in fact, partly explain the drilling effect seen with higher frequency as well as with direct contact with the stone. It stands to reason that power settings made with high frequency, and by default low Joules, may produce the insufficient ejection of materials and thus more resolidification resulting in more of a drilling effect in larger stones. The same principle likely explains the propensity for a probe to become impacted in a stone given that material not ejected, due to drilling, may resolidify behind the probe tip and prevent the removal of an impacted probe, which can be quite problematic. In these circumstances, we have found that forcefully pulling on the probe can help withdraw it from the stone, but this emphasizes the absolute need to avoid directly contacting the stone with the probe. Another issue arises when a laser probe drills through a stone and cannot be readily identified as being against the ductal epithelium due to loss of visibility of the probe tip. The periodic withdrawal of the probe in conjunction with saline irrigation through the cholangioscope can improve visibility while ensuring that the probe does not inadvertently target the bile duct.

For further adoption of LL to occur for the treatment of refractory bile duct stones, investigation of the cost-effectiveness of this technology is needed. From an accessibility standpoint, EHL has become widely utilized due to the relative ease with which a charge generator (Autolith Touch, Northgate Technologies Inc, Elgin, IL, USA) can be purchased (often as a package deal with the cholangioscopy system) and integrated into the endoscopy unit. In comparison, LL generators are costly, requiring either a large upfront capital cost to purchase or ad hoc renting of the LL system. If renting the LL system, cases must be planned beforehand, which can be problematic if LL is not actually utilized (e.g., a stone is able to be removed with standard maneuvers without the use of cholangioscopy-guided lithotripsy) or when performing a case during which LL is unexpectedly needed (e.g., impacted stone within an extraction basket). Furthermore, LL systems often require 220-volt electrical power, which may not be feasible in older endoscopy units and may require upgrading of the endoscopy rooms to accommodate this power level. Additionally, a laser technician is often required to operate the LL generator unlike EHL which can be operated by the endoscopist and standard endoscopy personnel. Nevertheless, LL can offer significant cost savings in potentially reducing the number of procedures and decreasing the number of fibers needed. Therefore, a randomized trial comparing LL with EHL will likely be needed to determine the true cost-effectiveness of LL in the management of large, refractory bile duct stones. Further investigation is also needed to determine the optimal timing of performing LL (during index procedure or subsequent procedures) to offer the maximum cost benefit to both the patient and medical center [26].

The limitations of this study include the use of explanted bile ducts that were devascularized and thus potentially prone to easier perforation, as well as the lack of surrounding connective tissue. These issues would provide skewed data in favor of safety within the parameters we observed, rather than opposite, lending more credence to the safety data shown here. We hypothesize that the method of directly placing the laser probe in contact with the bile duct by the operator would unavoidably present variability in tension and contact time with the epithelium, thus affecting the perforation rates. However, when performed in vivo, these exact conditions exist, as when contact is made with ductal epithelium, the tension produced and the time applied by the laser probe is variable between users and is not standardized. Another limitation remains the lack of testing within the intrahepatic ducts. Intrahepatic stones present a particular clinical challenge given their relatively narrow diameter in comparison to the common bile duct. In combination with their tortuous anatomy, this makes it difficult to advance devices such as an extraction balloon or basket to the level of the stone [3]. Furthermore, in conditions such as primary sclerosing cholangitis, these intrahepatic stones are often impacted due to the presence of a biliary stricture and can be difficult to identify under fluoroscopy alone [27]. Thus, cholangioscopy-guided lithotripsy would appear to offer an effective treatment option for these intrahepatic stones provided that the cholangioscope is able to be advanced to the level of the stone. Ex vivo studies, however, are lacking with regard to the safety of LL within the intrahepatic ducts where the risk of injury may be greater due to the narrow diameter of these ducts. Lastly, in this study we did not utilize the laser probe through a cholangioscope, instead directly holding the probe in proximity to the bile ducts and gallstones. While this allowed for the precise placement of the probe, this does not incorporate the challenge of performing LL through a cholangioscope. Orienting the probe can be challenging with the cholangioscope given that the probe comes out at the 6 o’clock position of the cholangioscope. Furthermore, once the probe is within the working channel of the cholangioscope, the cholangioscope itself is more difficult to maneuver, which further restricts the positioning of the laser probe. Patient movement during the ERCP can also affect the distance from the probe to the stone, which was not simulated in our ex vivo model. Nevertheless, by placing the probe directly perpendicular to the bile ducts in our study, we sought to determine the shortest amount of time needed to perforate the biliary epithelium to serve as a surrogate for the maximum burst period for laser use in the biliary tract.

The development of cost effective single-operator disposable cholangioscopes in recent years have made the delivery of EHL and LL laser under direct visualization of the bile duct much more readily available [14,28,29]. Several Ho:YAG laser lithotripsy systems have been FDA approved for managing difficult-to-treat bile duct stones [30]. Today, these exciting technologies are being used in challenging cases to avoid surgical interventions.

## 5. Conclusions

In summary, advances made in single-operator cholangioscopes have greatly enhanced the endoscopists’ ability to treat bile duct stones. While randomized data have demonstrated the efficacy of LL in particular in treating large, refractory bile duct stones, our data demonstrates that the Holmium-YAG laser is safe for use in the bile duct at power settings between 8–12 watts and at short bursts of 5 s or less. The effective fragmentation of bile duct stones is dependent upon the stone size and laser frequency, with best results occurring at lower frequencies. Optimal settings for stones larger than 2 cm are 12 watts at a low frequency such as 5 Hz. Improved fragmentation rates can be achieved in stones >2 cm by using a larger diameter (550 µm) laser probe. Caution should be taken not to “push” the probe through the stone due to increased risk of drilling and impacting the fiber, and potential ductal perforation. As a next step, we hope to expand this work to identify ideal operating parameters for lithotripsy of pancreatic duct stones, which present another set of challenges given the relatively narrow pancreatic duct and greater density (higher calcium content) of pancreatic duct stones.

## Data Availability

The data presented in this study are available from the corresponding author. The data are not publicly available currently as the data has yet to be deposited into a publicly available repository.
